# Systemic immune-inflammation index predicts the clinical outcomes in patients with acute uncomplicated type-B aortic dissection undergoing optimal medical therapy

**DOI:** 10.1186/s12872-023-03596-y

**Published:** 2024-01-02

**Authors:** Ruirong Chen, Sheng Su, Changjin Wang, Yuan Liu, Wenhui Huang, Songyuan Luo, Fan Yang, Jianfang Luo

**Affiliations:** 1 Department of Anesthesiology, Guangdong Provincial People’s Hospital (Guangdong Academy of Medical Sciences), Southern Medical University, Guangzhou, China; 2 Department of Cardiology, Guangdong Cardiovascular Institute, Guangdong Provincial People’s Hospital, Guangdong Academy of Medical Sciences, Southern Medical University, Guangzhou, China; 3 Department of Emergency and Critical Care Medicine, Guangdong Provincial People’s Hospital, Guangdong Academy of Medical Sciences, Southern Medical University, Guangzhou, China

**Keywords:** Type B Aortic Dissection, Inflammation, Optimal medical therapy, Aortic intervention, Composite outcomes

## Abstract

**Background:**

Optimal medical therapy (OMT) for uncomplicated type B aortic dissection (uTBAD) provides excellent short-term outcomes during follow up; however, its long-term therapeutic effectiveness is unsatisfactory. This study evaluated the predictive value of systemic immune-inflammation index (SII) for adverse events among patients with acute uTBAD undergoing OMT.

**Methods:**

We performed a retrospective analysis of a prospectively maintained database between 2013 and 2020. The primary end point in this study was composite outcomes including aortic intervention, all-cause mortality, retrograde type A aortic dissection (rTAAD) and aortic diameter growth > 5 mm. The patients were divided into high and low SII groups according to the optimal cut-off value of SII as determined using the receiver operating characteristic curve. Cox proportional hazards models were constructed to estimate the hazards ratios and identify the predictors of composite outcomes.

**Results:**

A total of 124 patients with acute uTBAD who underwent OMT were enrolled. One patient died during hospitalisation. At the end of a mean follow-up duration of 51 ± 23 months, 53 (43.1%) patients experienced composite outcomes, 15 patients (12.2%) died, 31 (25.2%) underwent aortic intervention, 21 (17.1%) exhibited diameter growth of > 5 mm, and 2 developed rTAAD. The patients were divided into low SII group (n = 78, 62.9%) and high SII group (n = 46, 37.1%) as per the optimal cut-off SII value of 1449. The incidence of composite outcomes in high SII group was significantly higher than that in low SII (28 [60.9%] vs. 26[33.3%], p < 0.01). Patients with high SII demonstrated significantly higher mortality rate than those with a low SII (11 [23.9%] vs. 5 [6.4%], respectively; p < 0.01). In addition, the high SII group had significantly higher rate of aortic-related reinterventions than the low SII group (16 [34.8%] vs. 15 [19.2%], p = 0.03). Multivariable Cox analyses showed that a high SII score was independently associated with composite outcomes rate (hazard ratio, 2.15; 95% confidence interval, 1.22–3.78; p < 0.01).

**Conclusions:**

The long-term therapeutic effectiveness of OMT alone in patients with acute uTBAD is unsatisfactory. An SII > 1449 at the time of diagnosis is an independent predictor of OMT failure.

**Supplementary Information:**

The online version contains supplementary material available at 10.1186/s12872-023-03596-y.

## Background

Optimal medical therapy (OMT) is the recommended treatment for patients presenting with uncomplicated type B aortic dissection (uTBAD). Although OMT is associated with excellent short-term prognosis, the long-term therapeutic effectiveness with OMT alone is unsatisfactory [1–3]. Emerging evidence has demonstrated favorable outcomes when thoracic endovascular aortic repair (TEVAR) is used to treat uTBAD, as it can promote aortic remodeling and prevent aortic dissection progression [4, 5]. Thus, it is essential to select those patients with uTBAD who would benefit from early endovascular therapy.

Multiple radiographic predictors of late aortic events have been identified, such as a large aortic diameter and patent or partially thrombosed false lumen [6]. However, few studies have focused on laboratory biomarkers that predict late aortic events. Inflammation is involved in several diseases, including chronic heart failure, cardiovascular diseases and aortic dissection (AD) [7, 8]. A high degree of inflammation predicts poor outcomes in AD [9].

Recently, the systemic immune-inflammation index (SII) was developed to account for the inflammatory and immune statuses of patients. A high SII is related to poor outcomes in patients with cardiovascular disease [10, 11]. For example, a high SII can predict the severity of stable coronary artery and ischemic stroke [12, 13]. However, few studies have examined the usefulness of SII in patients with acute uTBAD undergoing OMT. In this study, we aimed to determine the predictive role of SII in patients with acute uTBAD undergoing OMT.

## Methods

### Patient population

In total, 202 patients with uTBAD who were treated with OMT between 2013 and 2020 were retrospectively identified through hospital discharge lists from Guangdong Provincial People’s Hospital. Patients with complicated TBAD (cTBAD; n = 0), malignant tumor (n = 4), AD due to trauma, iatrogenic injury, or Marfan syndrome (n = 4), previous aortic intervention (n = 1), ascending aorta disease (n = 17), or subacute and chronic aortic dissection (n = 52) were excluded. Complicated TBAD is characterized by refractory pain, refractory hypertension, malperfusion syndrome, rupture and impending rupture. Uncomplicated TBAD does not lead to the abovementioned clinical features. The acute phase is defined as day 1–14 of onset of TBAD. The study received ethical approval from the Institutional Ethics Committee of Guangdong Provincial People’s Hospital. Informed consent from patients was not required due to the retrospective study design.

### Data collection

Electronic medical record was used to collect demographic data, comorbidities, laboratory findings and imaging features. Venous blood samples were obtained from all patients within the first 24 h after admission. The complete blood count was reviewed before discharge. The SII was calculated as total peripheral platelet count (P) × neutrophil-to-lymphocyte (N/L) ratio (SII = P × N/L ratio). Computed tomography (CT) images were analyzed using TeraRecon. The maximal aortic diameter was measured orthogonally to the vessel center line, which was automatically obtained by TeraRecon. The follow-up clinical outcome was determined from outpatient records or telephone interviews. The most recent CT findings during follow-up were compared with those at the time of disease onset. If the patient provided consent to aortic intervention during follow up, the final CT images before intervention were analyzed. In cases of death, every effort was made to determine the underlying cause. In cases of multiple composite outcomes in the same patient, we only recorded the time until the first event.

### Treatment protocol

According to current guidelines for AD, OMT was provided to all patients with acute uTBAD, which included strict control of blood pressure and heart rate during hospitalization. The target systolic blood pressure was 100–120 mmHg and the target heart rate was 60–70 beats/min. Beta-blockers were used routinely, unless contraindicated. Other antihypertensive drugs were also administered if needed, depending on the patients’ tolerance [14]. Pain killers were used to resolve the symptoms according to the clinical scenario, without any dose limitations. During the initial hospitalization, all patients were provided OMT and none underwent TEVAR.

The discharge criteria included blood pressure and heart rate within the target ranges and remission of symptoms.

### Clinical outcomes and operation indication

The primary end point of this study was composite outcomes including aortic intervention, all-cause mortality, retrograde type A aortic dissection (rTAAD) and aortic diameter growth > 5 mm. During follow-up, intervention was recommended when one of the following situations occurred: maximum aortic diameter (MAD) exceeded 5.5 cm, rapidly dilating aortic diameter (≥ 5 mm/year), aortic rupture, malperfusion, rTAAD and intractable pain [14]. The intervention was not performed until the aforementioned indications occurred and the patient provided consent.

### Statistical analysis

Data were shown as counts (with proportion as a percentage), means and standard deviations or median with interquartile ranges. Qualitative variables were compared using χ2 analysis, or Fisher’s exact tests, as appropriate. To analyze continuous variables, student t test and Mann–Whitney U test were used [15].

Receiver operating characteristic curve and the area under the curve were used to explore predictive value of admission SII with composite outcomes. Univariate Cox proportional hazards analysis was used to explore the associations between demographic characteristics, comorbidities, laboratory results, and imaging variables findings, and composite outcomes. To determine the independent risk factors for composite outcomes, multivariate cox regression models were used [15]. D-dimer levels exhibited a skewed distribution and were logarithm-transformed for further analysis. Variables with a p-value < 0.1 in the univariate analysis were entered into the multivariate analysis. To identify the independent predictors of composite outcomes, forward stepwise model selection was used. Survival and intervention rates were estimated using Kaplan-Meier curves. Furthermore, differences between groups were analyzed using the log-rank test. Statistical analyses were performed using SPSS version 25.0 (IBM Corp., Armonk, NY, USA). P values < 0.05 were considered statistically significant.

## Result

### Baseline characteristics

In total, 124 patients with acute uTBAD patients undergoing OMT were analyzed. The study participants included 81 (65.3%) men. The most common coexisting conditions among the patients were hypertension (n = 90, 72.6%), followed by smoking (n = 55, 44.4%). The mean maximum aortic diameter was 39 mm. Furthermore, 44 (35.5%) patients had a patent false lumen (FL), while 54 (43.5%) had partial thrombogenesis and 26 (21.0%) had thrombogenesis.

**Fig. 1:**
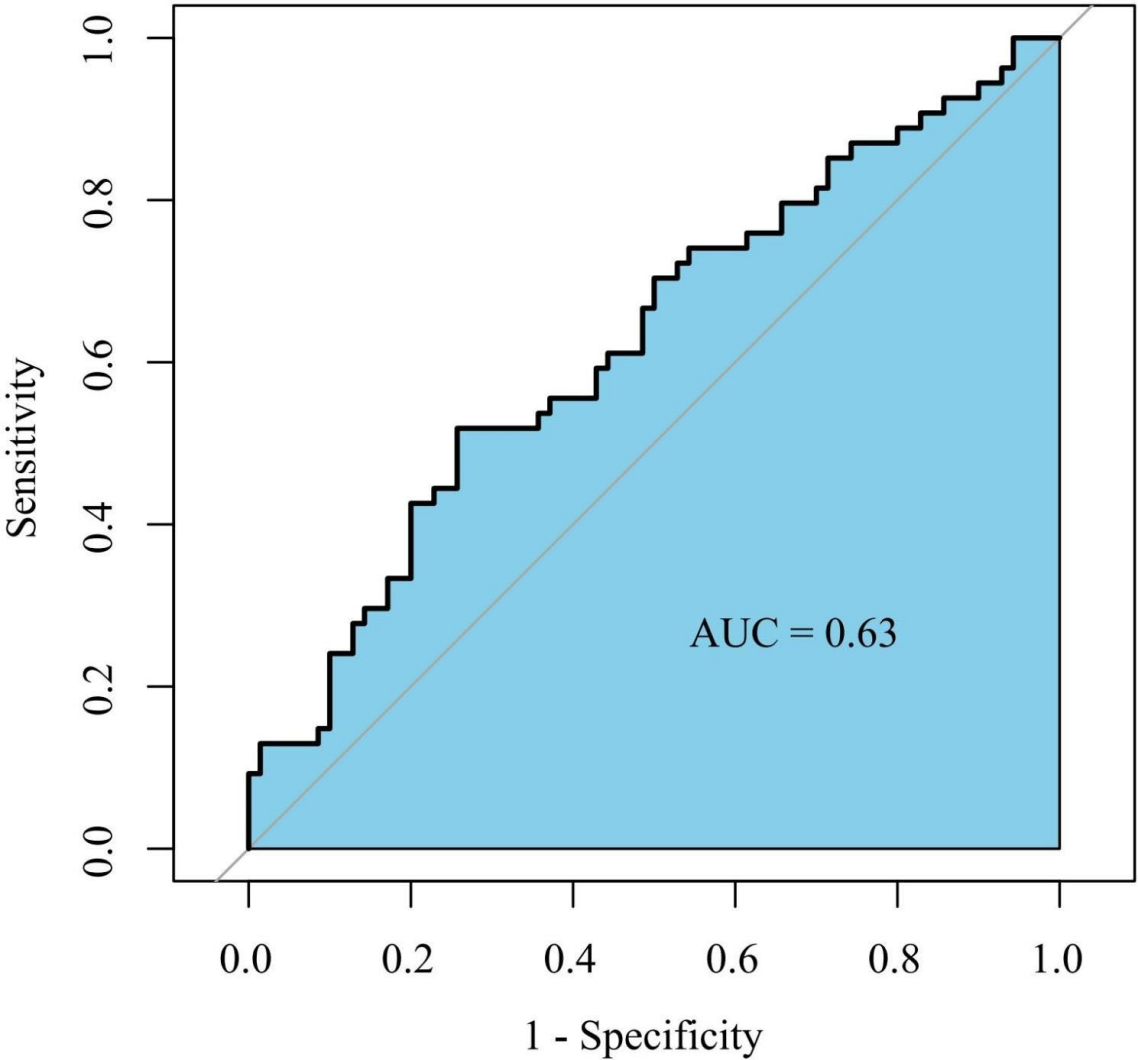
Receiver operating characteristic curve analysis with the area under the curve of SII index in predicting composite outcomes SII: Systemic immune-inflammation index

Receiver operating characteristic (ROC) curve was used to explore the predictive value of SII at admission with composite outcomes. The area under the curve (AUC) was 0.63 (95% confidence interval [CI], 0.53–0.73, p = 0.02) (Fig. 1). The optimal cut-off value of SII was 1449. According to the optimal cut-off value of SII, the patients were divided into low SII (≤ 1449, n = 78) and high SII (> 1449, n = 46) groups.

The baseline characteristics of the participants according to the SII score groups are described in Table 1. There were no differences in the age, sex, coronary artery disease, hypertension, chronic renal disease, cerebrovascular disease, MAD, extent of AD, or FL status between the two groups. In terms of oral medicine drugs at the time of discharge, the high SII group had a significantly higher proportion of beta blockers than the low SII group (41 [89.1%] vs. 58 [74.3%], respectively; p < 0.05). The platelet and neutrophil counts were significantly higher in the high SII group compared to the low SII group (260 ± 79 × 10^9^/L vs. 207 ± 82 × 10^9^/L, respectively, p < 0.01 and 10.7 ± 3.9 × 10^9^/L vs. 7.3 ± 2.5 × 10^9^/L, respectively, p < 0.01), while the lymphocyte count was significantly lower (1.2 ± 0.5 × 10^9^/L vs. 1.8 ± 1.2 × 10^9^/L, respectively; p < 0.01). In addition, the high SII group had a significantly higher level of C-reactive protein than the low SII group (p = 0.04) (Table 1). The SII value was significantly higher in the high SII group than the low SII group (2803 ± 2001vs. 885 ± 322, p < 0.01).

**Table 1:** Baseline demographics

	SII ≤ 1449(n = 78)	SII>1449(n = 46)	*P*
Age (years)	55 (48, 64)	55 (48, 66)	0.49
Gender, n (%)			0.23
male	54 (69.2)	27 (58.7)	
female	24 (30.8)	19 (41.3)	
Concomitant disorders, n (%)			
CAD	2 (2.7)	3 (6.5)	0.36
Hypertension	54 (69.2)	36 (78.3)	0.28
Diabetes	2 (2.7)	4 (8.7)	0.19
CKD	3 (3.8)	3 (6.5)	0.67
Cerebrovascular disease	5 (6.4)	2 (4.3)	1.00
Smoking	40 (51.3)	15 (32.6)	0.04
Laboratory results			
D-dimer	1890 (1200, 3800)	3650 (1870, 7115)	0.07
C reactive protein	69 ± 60	109 ± 88	0.04
PLT(×10*9/L)	207 ± 82	260 ± 79	0.01
NEUT(×10*9/L)	7.3 ± 2.5	10.7 ± 3.9	< 0.01
LYMPH(×10*9/L)	1.8 ± 1.2	1.2 ± 0.5	0.01
SII	885 ± 322	2803 ± 2001	< 0.01
Imaging results			
MAD in lesion (mm)	38 ± 9	40 ± 9	0.46
Debakey III			0.29
IIIa	16 (20.5)	6 (13.0)	
IIIb	62 (79.5)	40 (87.0)	
FL status			0.93
patent	27 (34.6)	17 (37.0)	
partial thrombogenesis	35 (44.9)	19 (41.3)	
thrombogenesis	16 (20.5)	10 (21.7)	
Oral medication for discharge			
β blocker	58 (74.3)	41 (89.1)	< 0.05
Alpha blocker	18 (23.1)	13 (28.3)	0.52
ACEI/ARB	61 (78.2)	34 (73.9)	0.59
CCB	56 (71.8)	36 (78.3)	0.43
hydragogue	26 (33.3)	11 (23.9)	0.27
statin	22 (29.2)	18 (39.1)	0.21

AAA: abdominal aortic aneurysm; PLT: platelet; NEUT: neutrophile; LYMPH: lymphocyte; FL: false lemon; MAD: maximum aortic diameter; CAD: coronary artery disease; CKD: chronic renal disease; ACEI/ARB: angiotensin converting enzyme inhibitors/ angiotensin II receptor blockers; CCB: calcium channel blocker

### Outcomes

In-hospital mortality occurred in one patient in the high SII group, which was caused by bacteraemia and massive gastrointestinal bleeding (Table 2). This patient was a 52-year-old man with DeBakey IIIb AD with a patent FL and MAD of 56 mm. There was no refractory pain, refractory hypertension, malperfusion syndrome, rupture, or impending rupture at the onset. Fungal cultures revealed the growth of Candida albicans. Despite the aggressive use of multiple antibiotics, the patient developed septic shock and subsequently died.

**Table 2:** Clinical outcomes

	SII ≤ 1449(n = 78)	SII> 1449(n = 46)	P
In-hospital			
In-hospital Time (days)	8 ± 5	10 ± 6	0.10
In-hospital Death	0	1(2.2%)	0.37
Follow-up (months)	52 ± 22	49 ± 23	0.51
Composite outcome	26 (33.3%)	27 (60.0%)	0.01
Death	5 (6.4%)	10 (22.2%)	0.01
Intervention	15 (19.2%)	16 (35.6%)	0.03
rTAAD	1 (1.3%)	1 (2.2%)	0.68
Diameter growth > 5 mm*	11 (17.2%)	10 (28.5%)	0.34

rTAAD: retrograde type A aortic dissection *: Patients who died or did not have reexamination CT during follow-up were excluded, last CT before intervention

**Fig. 2:**
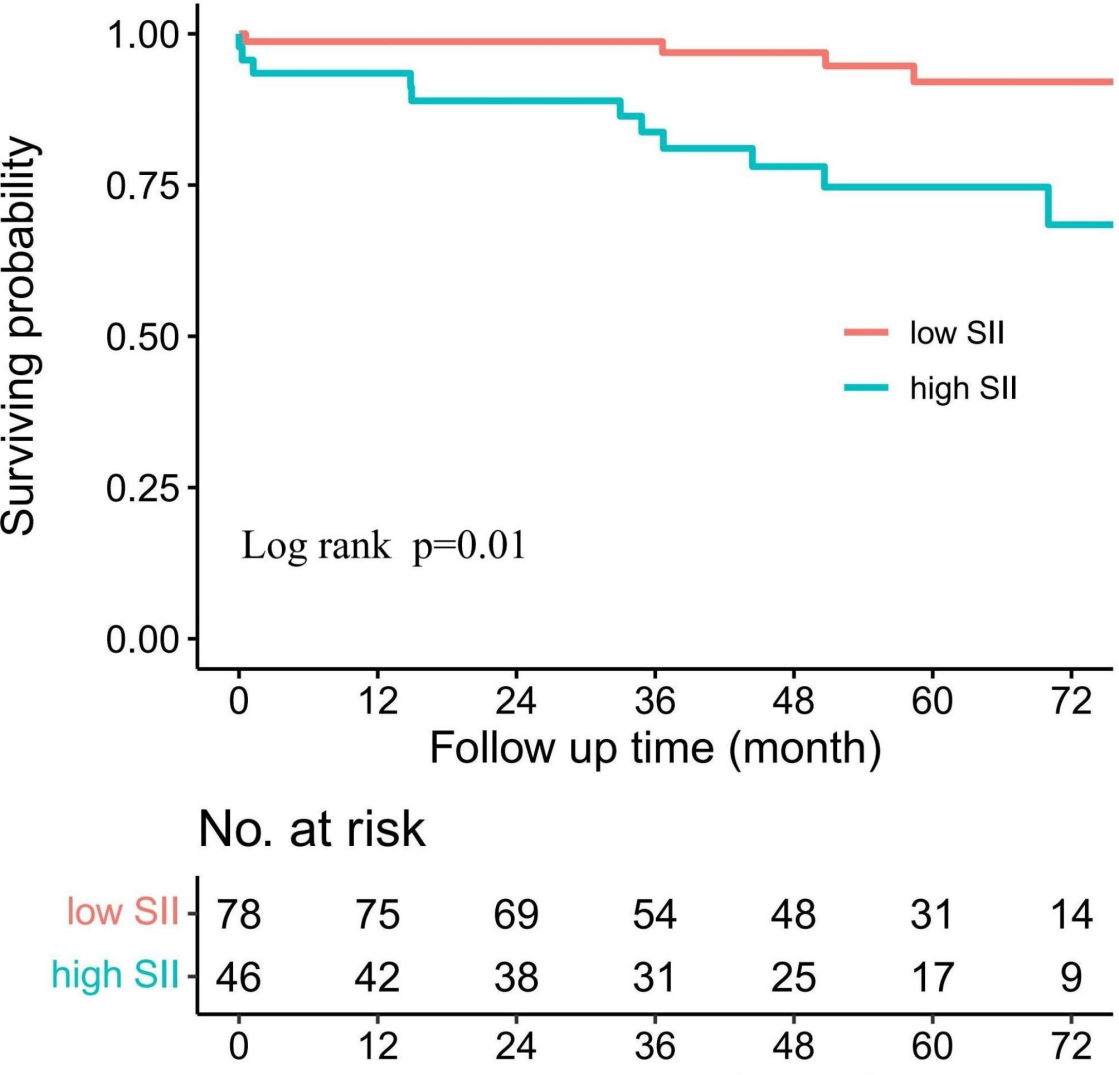
Kaplan-Meier survival curve analysis showed follow-up death by the SII index SII: Systemic immune-inflammation index

The mean follow-up duration was 51 ± 23 months and 53 (43.1%) patients experienced composite outcomes. Follow-up mortality occurred in 15 patients (12.2%) and aortic intervention was needed in 31 patients (25.2%) (Table 2). Among the 15 patients, 10 suffered aortic related death, 2 suffered non aortic related death and 3 had an unknown cause of death. In the high SII group, 10 patients died during follow-up, of which 8 experienced AD-related death, 1 experienced cerebral hemorrhage, and 1 were an unknown cause of death. In the low SII group, 5 patients died during follow-up, of whom 2 experienced AD-related death, 1 died of myocardial infarction, and 2 had an unknown cause of death. The follow-up mortality rate was significantly higher in the high SII group than the low SII group (10 [22.2%] vs. 5 [6.4%], respectively; p = 0.01; Fig. 2). Of note, patients in the high SII group demonstrated a higher rate of aortic related death than those in the low SII group (8 [17.8] vs. 2 [2.6%], respectively; p < 0.01]. Follow-up rTAAD and diameter growth > 5 mm occurred more frequently in the high SII group than the low SII group, although the difference was not statistically significant. Among the 31 patients undergoing aortic related intervention during follow-up, 28 were due to sudden severe chest pain, 2 were due to rapid enlargements of the aortic diameter, and 1 was due to rTAAD. Of note, patients in high SII group had a higher rate of aortic related intervention than those in the low SII group (16 [35.6%] vs. 15 [19.2%], respectively; p = 0.03). The incidence of follow-up composite outcomes in high SII group was significantly higher than that in low SII (27 [60.0%] vs. 26[33.3%], p = 0.01).

Follow-up CT images from 99 patients fulfilled the inclusion criteria and were analyzed (Supplementary Table  1). In the most recent follow up CT, the false lumen was patent in 23 patients (23.2%), partially thrombosed in 57 patients (57.6%), and completely thrombosed in 19 patients (19.2%). The mean MAD was 41 ± 11 mm, which was significantly larger than the initial MAD (*p* < 0.01). The follow up MAD in high-SII group was higher than in low-SII group (42 ± 11 mm vs. 41 ± 11 mm, p = 0.45). The rate of aortic diameter growth > 5 mm was higher in the high SII group than the low SII group (10 [28.5%] vs. 11 [17.2%], respectively; p = 0.34] (Table 2).

In the univariate cox regression analysis for composite outcomes, the variables with p < 0.1 included age, diabetes, chronic renal disease, D-dimer, MAD, thrombogenesis FL. Cox survival analysis revealed that SII > 1449 was associated with composite outcomes (unadjusted hazard ratio [HR] 2.08, 3.96; 95% CI, 1.21 − 3.58; p < 0.01). After adjusting for potential risk factors, SII > 1449 remained an independent predictor for composite outcomes (adjusted HR 2.15; 95%CI, 1.22–3.78; p < 0.01, Table 3). Cox survival analysis revealed that SII > 1449 was associated with all-cause mortality (unadjusted HR, 3.96; 95% CI, 1.37 − 11.40; p = 0.01).

**Table 3:** Univariate and multivariable cox regression analysis for composite outcome

Clinical variables	Univariate predictors		Multivariate predictors	
	HR (95%CI)	*p*	HR (95%CI)	*p*
Age	0.97 (0.95, 0.99)	0.01	-	-
Diabetes	3.87 (1.15, 13.03)	0.03	6.18 (1.74, 21.93)	< 0.01
CKD	2.87 (1.02, 8.03)	< 0.05	-	-
MAD	1.03 (1.00, 1.05)	0.02	1.04 (1.01, 1.06)	0.01
Thrombogenesis FL	0.18 (0.06, 0.59)	< 0.01	0.15 (0.05, 0.49)	< 0.01
SII > 1449	2.08 (1.21, 3.58)	< 0.01	2.15 (1.22, 3.78)	< 0.01
Ln (D-dimer)	1.28 (0.97, 1.68)	0.08	-	-

CKD: chronic renal disease; MAD: maximum aortic diameter; FL: false lemon; SII: systemic immune-inflammation index

### Pre-discharge SII variations

In this retrospective analysis, 84 patients underwent more than one complete blood count before discharge (Table 4). Defore discharge SII was lower than that on admission (1413 ± 901 vs. 1527 ± 1426, p = 0.45). In addition, before discharge SII was significantly higher in high-SII group than that in low-SII (2044 ± 1399 vs. 1141 ± 549, p < 0.01) (Table 4).

**Table 4:** Before discharge SII variation

	SII ≤ 1449(n = 54)	SII> 1449(n = 30)	P
Before discharge PLT (×10*9/L)	276 ± 112	343 ± 122	0.01
Before discharge NEUT (×10*9/L)	7.0 ± 2.1	6.6 ± 3.1	0.74
Before discharge LYMPH (×10*9/L)	1.8 ± 0.7	1.4 ± 0.5	< 0.01
Before discharge SII	1141 ± 549	2044 ± 1399	< 0.01

PLT: platelet; NEUT: neutrophile; LYMPH: lymphocyte; SII: systemic immune-inflammation index

### Sensitivity analysis

SII was analyzed as a continuous variable to explore the diagnostic accuracy of this index. After adjusting for potential risk factors, SII (as a continuous variable, per 100 increasing) remained an independent predictor for composite outcomes (adjusted HR, 1.03; 95%CI, 1.02–1.05; *p* < 0.01). In addition, C-reactive protein, a commonly used marker of systemic inflammation, was analyzed to explore its association with composite outcomes using the ROC curve. The AUC was 0.43 (95% CI, 0.36–0.58, p = 0.61). The neutrophil-to-lymphocyte ratio (NLR) was also analyzed to explore its association with composite outcomes using the ROC curve, which showed an AUC of 0.56 (95% CI, 0.45–0.66, p = 0.29).

## Discussion

There were three main findings of this study. First, among patients with acute uTBAD managed with OMT, there were significant rates of follow-up mortality and need for intervention. Second, a high SII was associated with an increased risk of mortality during the follow-up period. Third, multivariable cox analyses showed that a large MAD, diabetes and high SII were independent risk factors for composite outcomes. Our findings showed that SII can predict the follow-up outcomes in patients with acute uTBAD undergoing OMT. Therefore, SII could be considered a useful and relatively simple tool for stratification of the risk of poor outcomes and for facilitating management decisions.

Aortic dissection is a life-threatening disease caused by a tear in the intimal layer of the aorta, leading to separation between the intimal and medial layers of the aortic wall and false lumen formation [16]. It is reported that mechanical stretch-induced endoplasmic reticulum stress promotes smooth muscle cell apoptosis, inflammation, and degeneration, providing insight into thoracic aortic aneurysm/dissection formation and progression [17]. Antihypertensive therapy is the preferred medical treatment for AD, and timely endovascular therapy with a stent graft may be indicated for patients who have or may be at risk for complications [14]. Therefore, it is essential to identify patients who are at increased risk of future aortic events to determine the optimal candidates for prophylactic endografts. Previous investigations into the radiographic risk factors of poor outcomes in AD patients have focused on two characteristics of TBAD anatomy: aortic diameter and false lumen. Multiple studies have identified that an aortic diameter > 40 mm at the time of presentation is a risk factor for subsequent poor outcomes [18–20], which is similar to our results.

TBAD is associated with inflammation, and a high degree of inflammation predicts poor outcomes in AD [9]. Although certain inflammatory factors are related to a poor prognosis in AD, these markers alone may not be particularly useful. Thus, a multi-biomarker strategy that combines biomarkers across the pathobiological axes of inflammation may provide incrementally useful prognostic information for predicting aortic related adverse events. PET/CT can assess inflammation in blood vessels and a high uptake [18] F-FDG on PET/CT is demonstrated to correlate with an increased risk of AD rupture and progression [9]. However, it is time-consuming, inconvenient and expensive. Moreover, some hospitals are not equipped with PET equipment. These factors limit the use of PET in patients with AD. Inflammatory markers based on laboratory tests, such as routine blood count, are cheap, convenient, and suitable for clinical practice, especially for long-term regular follow-up of patients, instead of PET-CT.

Recently, SII (calculated as total peripheral platelets count (P) × neutrophil-to-lymphocyte ratio) was developed as a novel index to evaluate the inflammatory and immune status of patients. A high SII was related to poor outcome in patient with cardiovascular disease [10], [11]. For example, a high SII can predict the severity of stable coronary artery and even predict ischemic stroke in the future [12, 13]. Platelets, neutrophils and lymphocytes play important roles in the inflammatory state of AD. During this process, platelets activate the coagulation systems, thereby consuming clotting factors and producing a hypercoagulable state [21]. In addition, platelets are associated with neutrophil activation and promote lymphocyte migration into peripheral lymph nodes [22–24]. Neutrophils are important regulators of inflammatory responses and can secrete serine proteases, cathepsins and reactive oxygen intermediates, leading to endothelial cell damage, platelet aggregation and a hypercoagulable state [24]. Neutrophils are related to endothelial damage, hypercoagulability, and platelet aggregation [25]. Increased numbers of neutrophils are present in the vessel wall of patients with AD compared to the normal aortic vascular tissue [26]. Lymphocytes play an important role in producing cytokines and provoking cytotoxic cell death [27]. T lymphocytes can induce apoptosis in aortic vascular smooth muscle and stimulate the synthesis of matrix metalloproteinases [28]. Excessive apoptosis promotes aortic inflammation and degeneration [17].

Our results identified an association between high SII and increased long-term failure of OMT in patients with acute uTBAD. After blood pressure and heart rate regulation and symptomatic relief, the pre-discharge SII was lower than the admission SII, which may be a result of reduced inflammation severity. In addition, the pre-discharge SII was significantly higher in the high SII group than the low SII group. Therefore, the use of SII may be more useful than using individual cell counts, providing a more objective and comprehensive indicator of the inflammatory-immune state. In addition, we explored the associations of the C-reactive protein level and N/L ratio with composite outcomes, which demonstrated negative results.

Based on our results, SII is a useful, simple, and cost-effective tool for pre-OMT risk classification, especially for resource-poor areas. Patients with a high SII may benefit from more aggressive surveillance and treatment. TEVAR is highly effective in remodelling the aorta in the acute and subacute phases because of the compliant, elastic nature of a dissection flap, which is easily reapproximated to the outer aortic wall by the endograft. Hence, patients with acute uTBAD and a high SII may require pre-emptive TEVAR, given their significantly increased risk of poor outcomes during follow-up. Recently, statins were identified to have anti-inflammatory properties in cardiovascular disease. Therefore, they may also be used in patients with TBAD and a high SII to decrease inflammation and potentially improve outcomes. Of note, regular CT is essential for patients with acute uTBAD undergoing OMT, particularly those with a high SII, to promptly identify disease progression, such as rTAAD.

This study had limitations inherent to a retrospective analysis. First, the sample size was small and we did not explore the pathophysiological mechanisms. Second, this study is applicable to patients with acute uTBAD managed with conservative treatment, and the value of this index in other periods should be further explored. Third, complete blood count was not performed routinely during follow up, and some of the patients did not undergo imaging after OMT. The dynamic changes in SII were not analysed. In addition, because of the lack of an external cohort, external validation could not be provided. Further well-designed prospective clinical trials are needed to assess the predictive value of SII among patients with uTBAD.

## Conclusion

Patients with acute uTBAD and a high SII are at an elevated risk of poor outcomes during follow-up. SII may serve as a valuable tool for risk stratification before OMT, and patients with a high SII at the time of admission may benefit from closer follow-up or earlier intervention.

### Electronic supplementary material

Below is the link to the electronic supplementary material.

Supplementary Material 1

## Data Availability

The data underlying this article will be shared on reasonable request to the corresponding author.
