# Supplementary material for: Systemic immune-inflammation index predicts the clinical outcomes in patients with acute uncomplicated type-B aortic dissection undergoing optimal medical therapy
Source: BMC Cardiovasc Disord. 2024 Jan 2;24:7. doi: 10.1186/s12872-023-03596-y (PMC10763462; doi:10.1186/s12872-023-03596-y)
Supplement: Supplementary file 1 — Supplementary Material 1 [file 12872_2023_3596_MOESM1_ESM.docx]

Supplementary table. 1

Follw up CT data.

|  | SII≤1449（n=64） | SII＞1449（n=35） | P |
| --- | --- | --- | --- |
| MAD in lesion (mm) | 41 ±11 | 42±11 | 0.45 |
| FL status |  |  | 0.35 |
| patent | 10 (15.6%) | 9 (25.7%) |  |
| partial thrombogenesis | 40 (62.5%) | 17 (48.6%) |  |
| thrombogenesis | 14 (21.9%) | 9 (25.7%) |  |
